# Stereotactic body radiotherapy for treating lung cancer with a leadless pacemaker: A case report

**DOI:** 10.1097/MD.0000000000041808

**Published:** 2025-03-07

**Authors:** Kazuki Ishikawa, Kohei Fukuda, Ryo Hanai, Mitsuru Kurosaki

**Affiliations:** a Department of Radiation Therapy, Nara Prefecture General Medical Center, Nara, Japan; b Department of Radiology Division, Nara Prefecture General Medical Center, Nara, Japan.

**Keywords:** case report, leadless pacemaker, lung cancer, stereotactic body radiotherapy

## Abstract

**Rationale:**

Stereotactic body radiotherapy (SBRT) is a precise treatment modality for lung cancer, delivering high-dose radiation to tumors while sparing surrounding organs. However, because of their intracardiac placement and proximity to the chest radiation field, leadless pacemakers (LLPMs) pose unique challenges that are not fully addressed by the existing protocols for conventional pacemakers.

**Patient concerns:**

In this case study, we aimed to emphasize the importance of identifying LLPMs before initiating SBRT for lung cancer and to discuss the necessary adjustments in treatment planning needed to accommodate these devices.

**Diagnoses:**

An 81-year-old female with stage IA adenocarcinoma in the left lower lobe of the lung underwent SBRT.

**Interventions:**

During initial planning, the presence of an LLPM implanted in the right ventricle of the heart was overlooked. According to the original rotational arc therapy plan, 5 Gy of radiation would have been delivered to the pacemaker; therefore, a revised treatment plan using a fixed-beam multiport approach was adopted to avoid exposing the device to radiation.

**Outcomes:**

Pacemaker functionality was unaffected post-treatment, and the therapy was concluded without complications.

**Lessons:**

This case emphasizes the critical need for identifying LLPMs prior to treatment and the importance of tailored radiotherapy plans to prevent device malfunction. The increasing use of these devices necessitates adherence to guidelines which recommend cumulative radiation doses of <5 Gy. Consequently, a thorough patient history and meticulous imaging review are required since identifying LLPMs on computed tomography can be challenging. Furthermore, effective SBRT in patients with lung cancer and LLPMs requires careful planning to ensure safety and therapeutic success. This case provides valuable insights for radiation oncologists, advocating for diligent pretreatment evaluation and customized radiation strategies in the context of evolving cardiac implant technologies.

## 1. Introduction

Stereotactic body radiotherapy (SBRT) is an important treatment modality in the curative management of lung cancer. It delivers high-dose irradiation to the primary tumor with minimal damage to organs at risk. The effectiveness of SBRT has been demonstrated, particularly in early-stage non-small cell lung cancer, achieving significant tumor control and improved survival rates.^[1]^

Protocols for conventional pacemakers aim to minimize adverse interactions with radiotherapy.^[2–4]^ Recently, a newer technology, the intracardiac implanted leadless pacemakers (LLPMs), has become popular and increasingly used.^[5]^ However, this device poses new challenges because of the potential proximity of the radiation field and a lack of empirical evidence defining the tolerance and vulnerability of LLPMs to ionizing radiation.

Therefore, these devices should be considered before planning radiotherapy. Here, we present a case in which an LLPM was discovered after radiotherapy planning, necessitating a change in the irradiation procedure. We also discuss the appropriate treatment planning for patients with implanted LLPMs.

Informed consent was obtained from the patient and her family, and approval was obtained from the Ethics Committee of the hospital.

## 2. Case presentation

**Figure 1.**
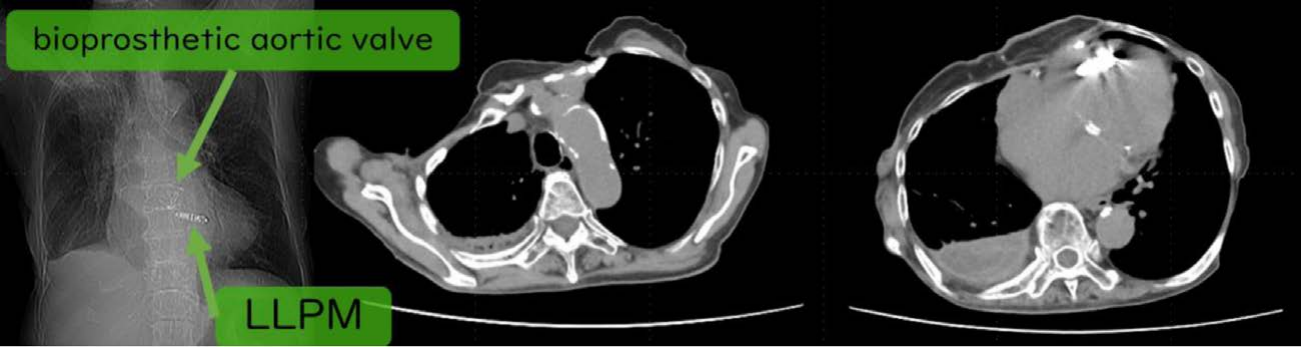
Computed tomography images showing no identifiable pacemaker or lead wires in the scout view or transverse images of the left anterior chest. A leadless pacemaker implanted in the patient’s right ventricle. LLPM = leadless pacemaker.

**Figure 2.**
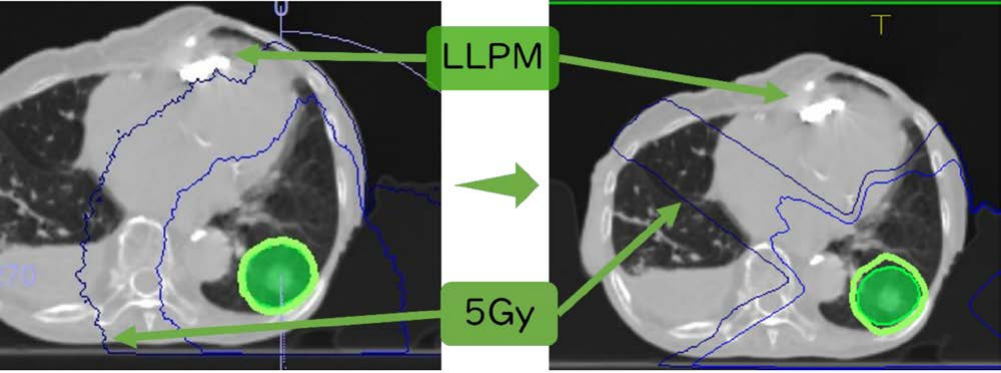
A leadless pacemaker (LLPM) implanted in the patient’s right ventricle. The left diagram shows the original treatment plan, indicating that 5 Gy would have been administered to the LLPM. The plan is revised to use a fixed multiport irradiation approach to avoid irradiating the LLPM (right). LLPM = leadless pacemaker.

An 81-year-old female was diagnosed with adenocarcinoma in the left lower lobe of her lung (cT1aN0M0, stage IA). The treatment plan included SBRT. During the initial consultation, the patient reported a history of complete atrioventricular block and pacemaker implantation; however, the type of device used was unknown. At that time, we were unaware of the presence of an LLPM. Although respiratory-synchronized computed tomography (CT) was performed to assist in treatment planning, the presence of the patient’s pacemaker was overlooked by both the CT technician and treatment planner (Fig. 1). Consequently, rotational arc therapy was planned. Subsequently, the patient was confirmed to have an LLPM implanted in her right ventricle. She had a history of surgery for right breast cancer, and replacing the LLPM with a leaded pacemaker was not anticipated at the time. According to the initial treatment plan, 5 Gy of radiation would have been delivered to the LLPM (Fig. 2). Therefore, a revised treatment plan using a fixed-beam multiportal irradiation approach was adopted to avoid exposure to the LLPM. Pacemaker assessments pre- and post-treatment revealed no abnormalities, and the treatment was completed without complications.

## 3. Discussion

This case emphasizes the importance of identifying the presence of LLPMs before conducting SBRT and the need for developing customized treatment plans to accommodate these devices. Malfunctions of pacemakers due to radiotherapy have been documented, prompting the establishment of treatment guidelines to avoid such issues.^[2–4]^ As LLPM use increases, the number of patients with these devices is expected to increase. Current guidelines advise precautions for patients with LLPMs similar to those recommended for traditional pacemakers. According to several user manuals, cumulative radiation doses of > 5 Gy may damage these devices (e.g., semiconductor element degradation and internal circuitry damage). In the case of a leaded pacemaker, it can be replaced on the opposite side. However, for LLPMs, removal is difficult, and an additional one is inserted when the corresponding years have passed.

Inquiring about a patient’s history of heart disease or pacemaker implantation is crucial during initial consultations. Traditional pacemakers are easily identified on CT scans of the left anterior chest region. However, LLPMs, which are implanted in the right ventricle, can be more difficult to detect and distinguish from other calcifications around the heart valves, potentially leading to oversights. Reports have indicated that treatment plans for patients with LLPMs have been modified in cases of lung cancer, breast cancer, and mediastinal lymphomas.^[6–8]^

Our institution typically employs rotational intensity-modulated radiotherapy to minimize treatment time. However, to avoid irradiating the patient’s LLPM, we used fixed multi-beam irradiation to ensure that the device was not irradiated.

In conclusion, this case study emphasizes the importance of identifying LLPMs before planning SBRT and the necessity of developing customized treatment plans to accommodate these devices. The insights gained from this case enhance our understanding and encourage a more nuanced approach when treating patients with such implants. Clinically, this knowledge empowers radiation oncologists to plan treatments carefully, ensuring patient safety and therapeutic efficacy in the complex landscape of modern cancer treatment.

## Author contributions

**Writing – original draft:** Kazuki Ishikawa.

**Writing – review & editing:** Kohei Fukuda, Ryo Hanai, Mitsuru Kurosaki.
